# 524 Traveling Pediatric Burn Care on Wheels

**DOI:** 10.1093/jbcr/irad045.121

**Published:** 2023-05-15

**Authors:** Mary Pruitt, Lydia Queale

**Affiliations:** UPMC Mercy Hospital, Venetia, Pennsylvania; UPMC Mercy Hospital, Pittsburgh, Pennsylvania; UPMC Mercy Hospital, Venetia, Pennsylvania; UPMC Mercy Hospital, Pittsburgh, Pennsylvania

## Abstract

**Introduction:**

Many burn patients initially seek treatment at a community hospital after suffering a burn injury and are then transferred to various hospitals before being evaluated by a burn specialist. Due to the increased volume of patients in the emergency department, pediatric burn patients in the past have waited on average 90 minutes to be assessed by a burn provider. A new process was implemented to decrease wait times and increase overall patient and family satisfaction. A breakdown in the process, noted by burn care staff, was the availability of burn care supplies located in the emergency department.

**Methods:**

• A process developed for burn unit nurses to respond to the emergency department to evaluate all pediatric burn patients was implemented.

• A survey was sent to burn nurses looking for the most used supplies while caring for a pediatric burn patient.

• Collaborative efforts with the pharmacy department to obtain space in the medication pyxis for the most used ointments in burn care.

• Collaborative efforts with the Child Life Specialist to stock the cart with stickers, small toys, and distraction items for pediatric patients.

• Collaborative efforts with the attending burn surgeon to develop and implement a protocol for burn team response.

• Collaborative efforts with infection control department to ensure proper regulation standards of the cart.

Cart Contents:

• Gauze

• Securement wraps

• Wound cleansers

• Silver impregnated foam dressing

• Non-stick petroleum gauze

• Staplers

• IV start supplies

• Paper tracking tool

• Discharge & dressing change instructions

• Stickers, small toys, & distraction items

**Results:**

The Hydro on Wheels cart was implemented in January 2022. A retrospective study of data including pediatric burn patients seen in 2021 was completed. The average wait time to be seen by a burn provider in the emergency department decreased from 90 minutes in 2021 to 34 minutes in the first 6 months of 2022.

A follow up survey was sent to hydrotherapy burn nurses to assess the satisfaction of the new process and equipment available.

**Conclusions:**

Overall decreased wait time for pediatric burn patients to be evaluated by a burn care provider.

Burn care nurses felt prepared when responding to a pediatric burn patient in the emergency department.

**Applicability of Research to Practice:**

The burn unit plans to continue this approach to improve patient and family satisfaction & patient outcomes

Future considerations to further improve the process would be to include a patient and family follow up satisfaction survey.

